# Efficacy and safety of transurethral resection of bladder tumour combined with chemotherapy and immunotherapy in bladder-sparing therapy in patients with T1 high-grade or T2 bladder cancer: a protocol for a randomized controlled trial

**DOI:** 10.1186/s12885-023-10798-2

**Published:** 2023-04-06

**Authors:** Xiangwei Yang, Shiqiang Zhang, Yajiao Cui, Yamei Li, Xinyue Song, Jun Pang

**Affiliations:** 1 Department of Urology, Kidney and Urology Center, Pelvic Floor Disorders Center, The Seventh Affiliated Hospital, Sun Yat-sen University, Shenzhen, China; 2 No.628 Zhenyuan Road, Shenzhen, 518107 China

**Keywords:** Bladder cancer, Chemotherapy, Immunotherapy, Transurethral resection of bladder tumour, Bladder-sparing therapy, Efficacy, Safety

## Abstract

**Background:**

Bladder cancer is the tenth most common cancer worldwide. For patients with T1 high-grade or T2 bladder cancer, radical cystectomy is recommended. However, radical cystectomy is associated with various complications and has a detrimental impact on the quality of life. Bladder-sparing therapy has been widely explored in patients with muscle-invasive bladder cancer, and whether a combination of transurethral resection of bladder tumour (TURBT) with chemotherapy and immunotherapy shows definite superiority over TURBT plus chemotherapy is still a matter of debate. The aim of this study is to investigate the efficacy and safety of TURBT combined with chemotherapy and immunotherapy in bladder-sparing therapy in patients with T1 high-grade or T2 bladder cancer who are unwilling or unsuitable to undergo radical cystectomy.

**Methods:**

An open-label, multi-institutional, two-armed randomized controlled study will be performed with 86 patients with T1 high-grade or T2 bladder cancer meeting the eligibility criteria. Participants in the experimental group (n = 43) will receive TURBT combined with chemotherapy (GC: gemcitabine 1000 mg/m^2^ on the 1st day and the 8th day, cisplatin 70 mg/m^2^ on the 2nd day, repeated every 21 days) and immunotherapy (toripalimab 240 mg on the 5th day, repeated every 21 days), and those in the control group (n = 43) will receive TURBT plus chemotherapy (GC). The primary outcome is pathological response, and the secondary outcomes include progression-free survival, overall survival, toxicities, and quality of life.

**Discussion:**

To the best of our knowledge, this is the first study to evaluate the efficacy and safety of TURBT combined with GC regimen and toripalimab in bladder-sparing therapy in patients with T1 high-grade or T2 bladder cancer. The expected benefit is that the combination of TURBT with chemotherapy and immunotherapy would be more effective than TURBT plus chemotherapy without compromising the quality of life and increasing the toxicity.

**Trial registration:**

ChiCTR2200060546, chictr.org.cn, registered on June 14, 2022.

## Background

Bladder cancer (BC) is the tenth most commonly diagnosed cancer and the thirteenth leading cause of cancer death worldwide, with an estimated 573 278 new cases and 212 536 deaths in 2020 according to GLOBOCAN estimates, accounting for 3.0% and 2.1% of all cancers, respectively [1]. The worldwide age-standardized incidence (per 100 000 person-years) is 9.5 in men and 2.4 in women, and the worldwide age-standardized mortality rate (per 100 000 person-years) is 3.3 for men and 0.86 for women [1]. Tobacco smoking and occupational exposure to aromatic amines, polycyclic aromatic hydrocarbons and chlorinated hydrocarbons are the two most important risk factors for BC [2]. Moreover, smoking is considered to have a detrimental impact on the efficacy of BC treatments and increase the risk of tumour recurrence and progression [3, 4].

Approximately 75% of BC patients present with a disease confined to the mucosa (stage Ta, carcinoma in situ) or submucosa (stage T1), which are grouped under the heading of non-muscle-invasive bladder cancer (NMIBC), and the remaining 25% of BC patients present with invasion of the detrusor muscle (stage T2), perivesical tissue (stage T3), and adjacent organs or tissues (stage T4) [5]. Urothelial carcinoma (UC) is the most common histology of BC, including pure urothelial carcinoma (PUC) and urothelial or nonurothelial histological variants (HVs) [6]. Most HVs show similar oncological outcomes after radical cystectomy (RC), but some of them, such as signet ring cell, spindle cell, and neuroendocrine tumours, are believed to have inferior survival compared with PUC [6]. The latest 2022 World Health Organization (WHO) classification of tumours of the urinary tract divides urothelial carcinomas into papillary urothelial neoplasm of low malignant potential (PUNLMP), non-invasive papillary carcinoma low grade (LG) and high grade (HG) [7], which is in accordance with the 2004/2016 classification system that provides better reproducibility than the older 1973 classification and is currently recommended worldwide [8, 9].

Transurethral resection of bladder tumour (TURBT) is an essential procedure for the complete removal of visible lesions and the correct diagnosis of BC, which requires the presence of detrusor muscle in the specimen and helps obtain the stage and grade of the cancers. A high percentage of patients with stage Ta T1 BC experience recurrence after TURBT; therefore, it is necessary to consider adjuvant treatment [10]. An immediate postoperative intravesical instillation of chemotherapy is one of the optimal choices due to its benefit in reducing the recurrence rate compared to TURBT alone [11].

For patients with muscle-invasive bladder cancer (MIBC), RC is the standard treatment [12]. In addition, RC is also recommended for patients with a high risk of recurrence and progression, including those with T1 HG BC, which is assessed by the European Association of Urology (EAU) NMIBC prognostic factor risk groups [13], because a delayed RC performed at the moment of progression to MIBC is correlated with a worse prognosis [14]. The recurrence-free survival and overall survival (OS) of MIBC patients undergoing RC were reported to be 68% and 66% at five years and 60% and 43% at ten years, respectively [15]. However, the benefit of RC must be weighed against its risks, impact on quality of life (QoL) and patient preference and tolerance. The early complications of RC, mainly consisting of infectious, genitourinary, gastrointestinal and wound related complications, occur in as many as 58% of MIBC patients [16], and the perioperative mortality was reported as 3.2% at 30 days and 5.2% at 90 days [17]. The QoL after RC is comparable after either continent or incontinent urinary diversion [18, 19], but both urinary and sexual function remain inferior to the general population [18].

Bladder-sparing therapy has been widely explored in patients with MIBC [20–24]. However, nearly 50% of patients who receive TURBT still need to undergo RC for recurrent MIBC with a disease-specific mortality rate of up to 47% [20], and radical radiotherapy has shown less OS benefit than radical surgery [21]. The combination of TURBT with systematic chemotherapy demonstrated that long-term survival could be achieved in a subset of MIBC patients, but the OS was still significantly lower than that of patients treated with RC, and the best candidates for this approach remain unclear [22]. Trimodal therapy (TMT) that combines TURBT, chemotherapy, and radiotherapy showed conflicting results compared to RC in patients with MIBC [23, 24]. Williams et al. revealed that TMT was associated with significantly decreased OS and cancer-specific survival [23], while Kulkarni et al. suggested that the survival outcomes were similar between patients who received TMT and those who received RC, and TMT can be offered to selected patients [24].

The addition of the programmed death receptor ligand-1 (PD-L1) inhibitor atezolizumab to platinum-based chemotherapy as first-line treatment for patients with metastatic UC has prolonged the progression-free survival (PFS) [25], and neoadjuvant gemcitabine and cisplatin (GC) plus a programmed cell death protein-1 (PD-1) inhibitor (pembrolizumab or nivolumab) has been reported to be associated with improved pathologic downstaging and to be generally safe [26, 27]. Nevertheless, whether a combination of PD-1/PD-L1 inhibitor with chemotherapy shows survival superiority over chemotherapy in bladder-sparing therapy is still a matter of debate. A multicentre real-world retrospective study considered it safe to apply bladder-sparing therapy for MIBC patients who achieved a pathological complete response (pCR) after neoadjuvant treatments, in which the highest pCR was observed among patients who accepted neoadjuvant immunochemotherapy, but a longer follow-up is needed [28].

Hence, we designed this prospective randomized controlled trial to investigate the efficacy and safety of TURBT combined with chemotherapy and immunotherapy compared to TURBT plus chemotherapy for patients with T1 HG or T2 BC unwilling or unsuitable to undergo RC. We hypothesized that a combination of TURBT with chemotherapy and immunotherapy would be more effective than TURBT plus chemotherapy without compromising QoL and increasing toxicity.

### Methods/design

**Fig. 1:**
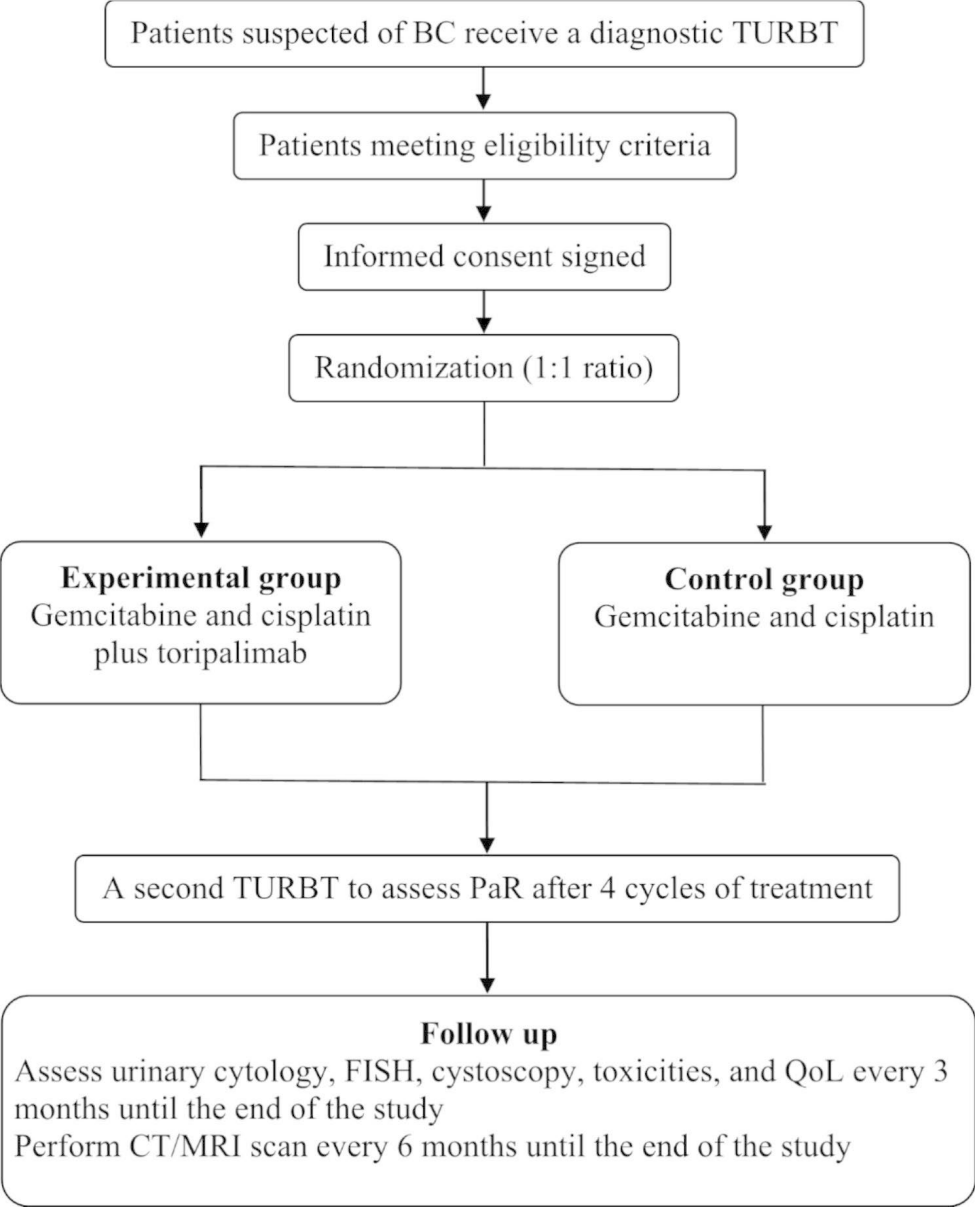
Flow chart of the study. BC: Bladder cancer; TURBT: Transurethral resection of bladder tumor; PaR: Pathological response; FISH: Fluorescence in situ hybridization; QoL: Quality of life; CT: Computed tomography; MRI: Magnetic resonance imaging

This is an open-label, multi-institutional, two-armed randomized controlled study. Patients admitted to the Seventh Affiliated Hospital of Sun Yat-sen University or the First Affiliated Hospital of Sun Yat-sen University suspected of having BC will be recruited. They will receive a diagnostic TURBT and an immediate postoperative intravesical instillation of chemotherapy, and those with pathological proof of T1 HG or T2 BC meeting the eligibility criteria will be randomly assigned to either the experimental group or the control group in a 1:1 ratio after informed consent is signed (Fig. 1).

### Inclusion criteria

- 18–75 years old
- Patients diagnosed with T1 HG or T2 BC unwilling or unsuitable to undergo RC.
- Patients with pathological proof of UC and radiographic proof of no metastasis of lymph nodes or distant sites.
- Patients who are fully informed of the aims and procedures of the study and sign the informed consent form.

### Exclusion criteria

- Any medical conditions that preclude them from chemotherapy, for example, allergy to chemotherapeutic drugs, performance status (PS) score > 1, glomerular filter rate ≤ 60 ml/min, degree of hearing impairment ≥ 2, peripheral neuropathy, grade III heart failure or above [29].
- History of any cancers other than BC.
- Combined with intravesical bacillus Calmette-Guérin immunotherapy, radiotherapy, or other antitumour treatments.
- Combined with autoimmune diseases or long-term use of immunosuppressive drugs (including prednisone > 10 mg/day).
- Contraindication to computed tomography (CT) or magnetic resonance imaging (MRI).
- Enrolled in another therapeutic trial.
- Any psychological, familial, sociological, or geographical condition hampering compliance with the study protocol and follow-up schedule.

## Outcomes

### Primary outcome

The primary outcome is pathological response (PaR), which includes pCR (from T2/T1 to T0) and pathological partial response (pPR: from T2 to T1/Ta, or from T1 to Ta). Pathological upstaging (from T1 to T2) and maintenance of pathological staging are defined as progression disease (PD) and stable disease (SD), respectively.

### Secondary outcomes

The secondary outcomes include PFS, OS, toxicity, and QoL.

- PFS is defined as the time from randomization until the first evidence of disease progression or death from any cause [30]. Radiographic progression will be evaluated according to the revised Response Evaluation Criteria in Solid Tumours (RECIST) guidelines (version 1.1) [31].
- OS is defined as the time from randomization to the date of documented death due to any cause [30].
- Toxicity will be assessed by the Common Terminology Criteria for Adverse Events (CTCAE) version 5.0 [32].
- QoL will be assessed by the European Organization for Research and Treatment of Cancer (EORTC) Quality of Life Questionnaire (QLQ-C30) [33].

### Sample size calculation

The sample size necessary for this trial was estimated based on the results of two previous studies [27, 34], whose PaR was 65.8% in patients treated with TURBT combined with chemotherapy and immunotherapy and 36% in patients treated with TURBT plus chemotherapy. We assumed 0 months as the superiority margin, and the randomization ratio was 1:1. We accepted a one-tailed type I error of 5% and type II error of 20%, and consequently, 68 patients needed to be recruited according to the computed result of PASS V.14 (NCSS, Kaysville, USA). Considering a maximum 20% dropout rate, a total of 86 patients will need to be included.

### Randomization

A randomized sequence (1:1 ratio) was generated by a clinical research assistant with SPSS Statistics V.23 (IBM, New York, USA). The study investigator will then receive a sealed envelope with an allocation sequence to randomize each participant.

### Blinding

Due to the nature of the intervention, it was deemed unrealistic to blind the study participants and clinical staff, which is why the study is not blinded.

### Interventions

After a diagnostic TURBT, patients randomized to the experimental group will receive chemotherapy (GC: gemcitabine 1000 mg/m^2^ on the 1st day and the 8th day, cisplatin 70 mg/m^2^ on the 2nd day, repeated every 21 days) plus immunotherapy (toripalimab 240 mg on the 5th day, repeated every 21 days), and those in the control group will receive chemotherapy alone (GC) (Fig. 1). Compared with the methotrexate, vinblastine, doxorubicin, and cisplatin (MVAC) regimen, the GC regimen provides a similar survival advantage but a better safety profile and tolerability [35]. Toripalimab is a humanized monoclonal antibody against PD-1 developed by Shanghai Junshi Bioscience Co., Ltd, and it was approved by the China National Medical Product Administration in 2021 for the treatment of UC due to its encouraging clinical activity and manageable safety profile [36–38]. After 4 cycles of treatment, all participants will be reexamined with urinary cytology and fluorescence in situ hybridization (FISH, a sensitive and specific test used to diagnose UC in urine) [39] and undergo a second TURBT to assess PaR. All participants will receive the standard of care, such as counselling of smoking cessation, to reduce the risk of tumour recurrence. A standard operating procedure will be developed in advance to provide step-by-step instructions to the staff on how to perform the study properly.

### Study visits

The study will be sustained for 5 years (from January 2023 to December 2027), during which specific milestones will be achieved and documented (Table 1). At each patient visit, the investigators will conduct history taking and physical examination and assess toxicities and QoL. Colony-stimulating factors will be used in case of grade 3 or 4 haematological toxic effects. After discontinuation of the protocol treatments, the participants will be followed up every 3 months until disease progression, death or the end of the study. Urinary cytology, FISH, and cystoscopy will be performed every 3 months, and chest and abdominal CT and pelvic MRI will be performed every 6 months. Additional examinations and treatments will be carried out at the discretion of the investigators.

**Table 1:** Timeline and follow-up of the study

	Screening	Treatment		Follow-up	
		Before every cycle	After 4 cycles	Every 3 months	Every 6 months
**Patient consent**	×				
**Demographics**	×				
**History & physical**	×	×	×	×	
**Blood cell counts**	×	×	×	×	
**Blood biochemistry**	×	×	×	×	
**FT3/FT4/TSH**	×	×	×	×	
**Cortisol/ACTH**	×	×	×	×	
**Urinary cytology**	×		×	×	
**FISH**	×		×	×	
**CT/MRI**	×				×
**Cystoscopy**	×		×	×	
**Toxicity assessment**		×	×	×	
**QoL**	×	×	×	×	

FT3: Free triiodothyronine; FT4: Free thyroxine; TSH: Thyroid-stimulating hormone; ACTH: Adrenocorticotropic hormone; FISH: Fluorescence in situ hybridization; CT: Computed tomography; MRI: Magnetic resonance imaging; QoL: Quality of life

### Data collection and management

A case report form has been prepared for data collection. Baseline data will be recorded based on medical records or specific worksheets, mainly including sociodemographic characteristics (age, gender, occupation, education level, etc. ), health characteristics (body mass index, PS score, signs and symptoms, QoL, history of previous treatments, smoking habits, comorbidities, etc. ), laboratory tests (blood cell counts, blood biochemistry, FT3/FT4/TSH, plasma cortisol/ACTH, urinary cytology, FISH, etc. ), and radiographic examinations (chest and abdominal CT and pelvic MRI). The pathological subtypes, staging and grading, expression of PD-L1 and human epidermal growth factor receptor 2 (HER-2), and EAU risk groups will also be recorded. Laboratory tests, radiographic examinations and cystectomy will be repeated during the follow-up as scheduled, as well as the assessment of QoL and tobacco use. All data will be cross-checked independently by two investigators, and the data on hard copies will be stored in a locked cabinet, while soft copies will be stored in an encrypted hard disk. All data collected will be used for research purposes only, and only the investigators will have access to the data. All data will be destroyed 5 years after the completion of the study.

### Statistical analyses

Two investigators will participate in the statistical process. Frequencies of events will be analysed using Pearson’s χ^2^ test or Fisher’s exact test, and continuous variables will be analysed by Student’s t test or nonparametric tests in case of a nonnormal distribution. The median PFS and OS will be estimated with the Kaplan-Meier method, and survival will be compared between groups using the log-rank test. Patients without progression, documented death or lost to follow-up at the time of analysis will be censored at the time of the latest assessment [40, 41]. A Cox proportional hazards regression model will be used to analyse the association between the survival time of the patients and the predictor variables, and the hazard ratio will be given with its 95% confidence interval. Missing data will be statistically analysed using the last observation carried forward method. All statistical analyses will be performed using Stata V.16 (StataCorp, Texas, USA). A two-tailed p value of 0.05 or less will be considered statistically significant.

### Data monitoring

A Data Safety Monitoring Committee (DSMC) consisting of two clinicians knowledgeable in the field of BC treatment, a statistician, a pharmacologist, and an ethicist will be set up to oversee the conduct of the study and to manage any data or safety issues that may arise. The DSMC members will not be directly involved in the conduct of the trial, and a DSMC meeting will be held at least once every 6 months. In any case of unacceptable toxicity, the DSMC can recommend early termination of the trial, and the investigator will decide whether the DSMC recommendations will be followed.

## Discussion

As mentioned above, RC is still the recommended treatment for patients with T1 HG or T2 BC [12, 13], therefore we confine eligible participants to those who are unwilling or unsuitable to undergo RC. We do not aim at comparing the survival benefit of bladder-sparing therapy over radical surgery, which will be examined in subsequent studies if satisfactory outcomes are achieved in this study. In the cases of failure of bladder-sparing therapy or intolerant toxicities or patients’ preferences change, an immediate RC will be discussed with patients.

Immune-related adverse events (irAEs) mainly involve the skin, liver, colon, endocrine glands, lung, and musculoskeletal system, leading to diseases such as dermatitis, pruritus, hepatitis, colitis, hypophysitis, hypothyroidism, hyperthyroidism, type 1 diabetes, pneumonitis, sarcoidosis, and inflammatory arthritis, etc. [42]. Most irAEs are mild to moderate in severity [42], and extensive clinical use of PD-1/PD-L1 inhibitors has proved to be generally safe [25–27]. Toripalimab is a PD-1 inhibitor widely used in China for the treatment of various cancers [36], with satisfactory anti-tumor effects and was generally well-tolerated [36–38]. We will closely monitor the potential side effects for early recognition and prompt intervention, and we expect to achieve a better therapeutic effect using the immunochemotherapy regimen without compromising QoL and increasing toxicity. If proved effective, the bladder-sparing immunochemotherapy regimen can be examined in patients with stage T3 or T4 BC or those with metastasis of lymph nodes who are also unwilling or unsuitable to receive RC.

This study firstly evaluates the efficacy and safety of TURBT combined with GC regimen and toripalimab in bladder-sparing therapy in patients with T1 HG or T2 BC. We record and track the tobacco use of the patients to know their changes in smoking behavior, the information may help us take appropriate measures to promote smoking cessation. The expression of PD-L1 in BC patients has a close relationship with a better objective response rate when compared with PD-L1 negative patients treated with toripalimab in previous studies [37, 38], and we record PD-L1 status to further explore the association. We also record the expression of HER-2 because a novel humanized anti-HER-2 antibody conjugated with monomethyl auristatin E, which is called RC48-ADC, has demonstrated a promising efficacy with a manageable safety profile in HER-2 + locally advanced or metastatic UC who had failed at least one line of systemic chemotherapy [43]. For those who fail bladder-sparing chemotherapy or immunochemotherapy and refuse to undergo radical surgery, RC48-ADC is a potential choice that may make a difference.

## Data Availability

Data sharing is not applicable to this article as no datasets were generated or analysed during the current study.

## Abbreviations

BC: Bladder cancer
NMIBC: Non-muscle-invasive bladder cancer
UC: Urothelial carcinoma
PUC: Pure urothelial carcinoma
HV: Histological variant
RC: Radical cystectomy
WHO: World Health Organization
HG: High grade
TURBT: Transurethral resection of bladder tumour
MIBC: Muscle-invasive bladder cancer
EAU: European Association of Urology
OS: Overall survival
QoL: Quality of life
TMT: Trimodal therapy
PD-L1: Programmed death receptor ligand-1
PFS: Progression-free survival
GC: Gemcitabine and cisplatin
PD-1: Programmed cell death protein-1
pCR: Pathological complete response
PS: Performance status
CT: Computed tomography
MRI: Magnetic resonance imaging
PaR: Pathological response
pPR: Pathological partial response
PD: Progression disease
SD: Stable disease
RECIST: Response Evaluation Criteria in Solid Tumours
CTCAE: Common Terminology Criteria for Adverse Events
EORTC: European Organization for Research and Treatment of Cancer
QLQ: Quality of Life Questionnaire
MVAC: Methotrexate, vinblastine, doxorubicin, and cisplatin
FISH: Fluorescence in situ hybridization
FT3: Free triiodothyronine
FT4: Free thyroxine
TSH: Thyroid-stimulating hormone
ACTH: Adrenocorticotropic hormone
HER-2: Human epidermal growth factor receptor 2
DSMC: Data Safety Monitoring Committee
irAEs: Immune-related adverse events
